# Characterization of cephalosporin and fluoroquinolone resistant Enterobacterales from Irish farm waste by whole genome sequencing

**DOI:** 10.3389/fmicb.2023.1118264

**Published:** 2023-03-22

**Authors:** Deirdre M. Prendergast, Rosemarie Slowey, Catherine M. Burgess, Declan Murphy, Dayle Johnston, Dearbháile Morris, Áine O’ Doherty, John Moriarty, Montserrat Gutierrez

**Affiliations:** ^1^Department of Agriculture, Food and the Marine, Celbridge, Co. Kildare, Ireland; ^2^Teagasc Food Research Centre, Ashtown, Dublin, Ireland; ^3^Antimicrobial Resistance and Microbial Ecology Group, University of Galway, Galway, Ireland

**Keywords:** antimicrobial resistance, cephalosporin, fluoroquinolone, Enterobacterales, environment, whole genome sequencing

## Abstract

**Background:**

The Enterobacterales are a group of Gram-negative bacteria frequently exhibiting extended antimicrobial resistance (AMR) and involved in the transmission of resistance genes to other bacterial species present in the same environment. Due to their impact on human health and the paucity of new antibiotics, the World Health Organization (WHO) categorized carbapenem resistant and ESBL-producing as critical. Enterobacterales are ubiquitous and the role of the environment in the transmission of AMR organisms or antimicrobial resistance genes (ARGs) must be examined in tackling AMR in both humans and animals under the one health approach. Animal manure is recognized as an important source of AMR bacteria entering the environment, in which resistant genes can accumulate.

**Methods:**

To gain a better understanding of the dissemination of third generation cephalosporin and fluoroquinolone resistance genes between isolates in the environment, we applied whole genome sequencing (WGS) to Enterobacterales (79 *E. coli*, 1 *Enterobacter cloacae*, 1 *Klebsiella pneumoniae*, and 1 *Citrobacter gillenii*) isolated from farm effluents in Ireland before (*n* = 72) and after (*n* = 10) treatment by integrated constructed wetlands (ICWs). DNA was extracted using the MagNA Pure 96 system (Roche Diagnostics, Rotkreuz, Switzerland) followed by WGS on a MiSeq platform (Illumina, Eindhoven, Netherlands) using v3 chemistry as 300-cycle paired-end runs. AMR genes and point mutations were identified and compared to the phenotypic results for better understanding of the mechanisms of resistance and resistance transmission.

**Results:**

A wide variety of cephalosporin and fluoroquinolone resistance genes (mobile genetic elements (MGEs) and chromosomal mutations) were identified among isolates that mostly explained the phenotypic AMR patterns. A total of 31 plasmid replicon types were identified among the 82 isolates, with a subset of them (*n* = 24), identified in *E. coli* isolates. Five plasmid replicons were confined to the *Enterobacter cloacae* isolate and two were confined to the *Klebsiella pneumoniae* isolate. Virulence genes associated with functions including stress, survival, regulation, iron uptake secretion systems, invasion, adherence and toxin production were identified.

**Conclusion:**

Our study showed that antimicrobial resistant organisms (AROs) can persist even following wastewater treatment and could transmit AMR of clinical relevance to the environment and ultimately pose a risk to human or animal health.

## Introduction

The Enterobacterales are a group of ubiquitous Gram-negative bacteria of increasing concern since they are human pathogens and can also transfer AMR genes to other pathogenic bacteria of the same and differing species *via* MGEs (Meek et al., 2015; Ruh et al., 2019; Arnold et al., 2022). Notably, transmissible resistance to the third generation cephalosporins has become a significant issue due to mounting levels of resistance in hospitals and community settings, which in turn can lead to severe infection and death (Jeanvoine et al., 2020).

WHO recognized and prioritized AMR bacteria into critical, high and medium based on the urgency and need for new antibiotics (WHO, 2019). Ireland, like many other countries has adopted a one health approach to address the challenge of AMR and published the second revision of Ireland’s National Action Plan on AMR in 2021 (Government of Ireland, 2021).

Although limited attention has been given to AMR in isolates from the environment in the past (Singer et al., 2016; Ramovic et al., 2020), EFSA recently produced a scientific opinion on the role played by the environment in the emergence and spread of AMR through the food chain, which identified a large number of data gaps in relation to the sources of contamination, relevance of transmission routes and effectiveness of mitigation measures (EFSA Biological Hazards (BIOHAZ) panel, 2021). It was concluded that there is insufficient data available to support a specific assessment of the quantitative impact that the production environment has on the contamination of foods or on public health due to limited studies on the efficiency of mitigation options for resistant organisms and elimination of resistance genes (EFSA Biological Hazards (BIOHAZ) panel et al., 2021). Antimicrobial resistant organisms (AROs) and MGEs can spread through several different pathways in the environment and have been previously reported in surface waters, soils, animal and human wastewater and foods (Graham et al., 2019). Molecular mechanisms for resistance can be either chromosomal or plasmid mediated, and this impacts on the transmissibility of the genes. The spread of AMR *via* MGEs such as plasmids, insertion sequences, integrons and transposons plays an important role in antibiotic resistance (Partridge et al., 2018). Among the molecular mechanisms for resistance to extended spectrum cephalosporins (alone or in various combinations) are the presence of genes encoding for TEM, ACT, CMY, SHV, OXA and CTX-M type β-lactamases (De Angelis et al., 2020; Hussain et al., 2021). Molecular mechanisms for chromosomal fluoroquinolone resistance involve mutations in target genes of the quinolone resistance-determining regions (QRDR) such as DNA gyrase (*gyr*A) or topoisomerase IV (*par*C or *par*E) or acquisition of plasmid mediated resistance such as the *qnr* gene and *oqx*AB, a quinolone efflux pump (Perez et al., 2013; Azargun et al., 2020; Chen et al., 2020). Characterization of AMR genes by PCR, WGS and a combination of PCR and metagenomics has previously been successfully applied to *E. coli* isolates from hospital wastewater, grassland soils and fecal samples, manure pits on dairy farms and from the environment and feces on broiler farms (Chandran et al., 2014; Yang et al., 2020; Massé et al., 2021; Byrne et al., 2022). The study of Massé et al. (2021) examined *E. coli* isolated from fecal samples from calves, cows and manure pits at 101 diary farms in Canada and reported that 85% of farms had at least one AmpC and ESBL producing *E. coli* and that *bla*_CMY-2_ and *bla*_CTX-M_ were the genes, respectively, responsible for these phenotypes. The study of Byrne et al. (2022) investigated the prevalence and frequency of ESBL/AmpC producing *E. coli* isolated from the environment and fecal samples from broiler farms in Ireland and reported that 13% of *E. coli* isolates from broiler farms harbored the *bla*_CMY-2_ gene and that hatcheries may be a reservoir and major contributor to the transmission of AMR.

Animal waste can be an important source of AMR bacteria entering the environment, especially if spread on soils where resistance genes can accumulate (Xu et al., 2020; Yang et al., 2020). We recently reported the identification of critically important Enterobacterales in wastewater from pig, cattle and poultry farms, both before and after treatment using ICWs (Prendergast et al., 2022a). Here, we report on the characterization of the isolates collected from farm wastewater before and after ICW treatment, AMR genes, pathotypes, phylogroups, plasmid replicons and virulotypes, and on the relatedness of the *E. coli* isolates, in order to enhance our understanding of the significance of farm effluents and ICW-treated water as a source of AROs to the environment in Ireland.

## Materials and methods

### Selection of isolates

All isolates collected from pre and post water treatment by ICWs in a previous study (Prendergast et al., 2022a) were sequenced. They included 72 that were isolated directly from the farm effluent at four farms, i.e., beef (120 sucklers; farm 1), dairy (100 lactating cows; farm 2), dairy and poultry (100 lactating cows and 8,000 broilers; farm 3) and piggery (1,590 sows; farm 4) and 10 from the ICW treated water (one isolate from farm 2 and nine from farm 3). All four farms were located in the southeast region of Ireland (Co. Waterford). Isolates were obtained using selective media to screen for the presence of carbapenem, cefotaxime (ESBL/pAmpC) and fluoroquinolone resistant organisms as described previously (Prendergast et al., 2022a). Among these isolates, the majority (*n* = 79) were identified as *E. coli*. The remaining three were *Citrobacter gillenii*, *Enterobacter cloacae*, and *Klebsiella pneumoniae*. Isolates were stored at −80°C in Protect beads (Langanbach services Ltd., Wicklow, Ireland) pending WGS.

### Whole genome sequencing (WGS)

Isolates were recovered on Columbia Agar supplemented with horse blood (E & O Laboratories LTD, Scotland, United Kingdom) and incubated at 37°C for 18–24 h. Using a 1 μL inoculation loop, a loopful of pure culture was taken from the plate and re-suspended in 100 μL nuclease free water and DNA was extracted and quality checked as previously described using the MagNA Pure 96 system (Roche Diagnostics, Rotkreuz, Switzerland) (Prendergast et al., 2022b).

Sample libraries for all isolates were prepared using the Illumina DNA Prep kit (Illumina, Eindhoven, Netherlands) The library was amplified by 5 PCR cycles and normalization, denaturing and sequencing of libraries was as previously described (Prendergast et al., 2022b). The isolates included in this study were distributed over eight sequencing runs on a MiSeq (Illumina).

### Analysis of WGS data

The following run metrics were used to check that the run passed basic quality metrics for raw sequence data, i.e., >70% bases higher than Q30 at 2 × 300 bp and cluster density of 1,100–1,400 k/mm^2^. In addition, the percentage of reads that aligned to the *phi*X was also checked to ensure that the starting concentration of the libraries were not over or under-estimated. The generated raw sequence reads (FASTQ files) were imported directly from Illumina BaseSpace to BioNumerics (Version 8.1; Applied Maths NV, a BioMérieux company, Sint-Martens-Letem, Belgium). FASTQ files were assembled using the SPAdes assembler within the BioNumerics software *via* its integrated calculation engine as previously described (Prendergast et al., 2022b). The FASTQ files were submitted to the BioNumerics wgMLST scheme for assembly-free calling and assembled genomes were submitted to the scheme for assembly-based allele calling. The sequence quality of each individual genome was evaluated using BioNumerics to include the following information: Number of contigs, N50, coverage, genome length, and core genome (%).

All genomes passed the basic quality metrics for raw sequence data from the Miseq. On average, a cluster density of 1,123 (K/mm^2^) was achieved with 91.23% of clusters passing filter (PF) specifications. Over the eight runs, the average number of reads, yield and error rate was 25,164,438 reads PF, 14.56 Gbp and 2.58% error, respectively. In each run, the index reads were evenly distributed across all samples.

A number of different knowledgebases within the *E. coli* plugin (V2021.04.12) in BioNumerics, were used to obtain data on acquired resistance, mutational resistance, acquired virulence, predicted pathotype and plasmids (ori-identity). The rules used in defining particular pathotypes and virulence within BioNumerics were as previously described (EFSA Biological Hazards (BIOHAZ) panel et al., 2020; Malberg Tetzschner et al., 2020). The plugin detected the pathotype based on the presence of certain marker genes as defined by the National Reference Laboratory at the Statens Serum Institute, Denmark. Possible pathotypes were enteropathogenic *E. coli* (EPEC), enterohemorrhagic *E. coli* (EHEC), enterotoxigenic *E. coli* (ETEC), enteroaggregative *E. coli* (EAEC), diffusely adherent *E. coli* (DAEC), enteroinvasive *E. coli* (EIEC), uropathogenic *E. coli* (UPEC), and neonatal meningitis *E. coli* (NMEC). APEC pathotypes were identified when isolates had three or more of the virulence genes *iss*, *iro*N, *hly*F, *omp*T, and *uit*A (Johnson et al., 2008). *E. coli* phylotyping was performed *in silico* by exporting Fasta files from BioNumercs and then uploading them to the CleroTyping research center^1^ without adjusting the settings (Beghain et al., 2018; Clermont et al., 2019).

The sequence types (STs) of the *E. coli* isolates were determined following the MLST scheme of Achtman, i.e., the 7 gene allele profile was determined from the sequence. In addition to the 7 housekeeping genes, BioNumerics enabled the analysis of the entire core genome (cgMLST) and phylogeny was inferred by creating a dendrogram with a scaling factor of 1 using the single linkage algorithm within BioNumerics. In the context of the diversity amongst the isolates examined, clusters, or matches by cgMLST were defined as a distance measure of ≤8 alleles. To determine the MLST of non-*E. coli* isolates, FASTQ were uploaded to the Center for Genomic Epidemiology (CGE)^2^ to extract information on MLST using MLST 2.0 (Larsen et al., 2012). Dendrograms of cgMLST were created in BioNumerics using the single linkage algorithm with the allele calls considered categorical data.

## Results

The *de novo* assemblies consisted of an average of 142 contigs (range 56–373 bp) with an average N50 of 162,544 bp (range 66,468–406,961 bp). The average coverage was 88X (range 40–135 X) and core % ranged from 98.8–100 with a mean core percent of 99.8%.

### Genotypic confirmation of AMR phenotypic results

Genotypic findings largely corresponded with phenotypic AMR results. Amongst the 48 isolates phenotypically identified as cefotaxime (CTX) resistant, 45 harbored genes encoding CTX-M-14 (*n* = 19), CTX-M-15 (*n* = 9), CMY-2 (*n* = 6), CTX-M-65 (*n* = 1) or SHV-12 (*n* = 1) or carried a mutation in the *amp*C promotor at position 12 (*n* = 9) and were therefore confirmed through WGS (Table 1). The *E. cloacae* isolate which was phenotypically described as ceftazidime (CAZ) resistant and CTX susceptible (MIC = 2 μg/mL) harbored *bla*_ACT-14_ and *bla*_SHV-12_ genes.

**Table 1:** Distribution of genes associated with cefotaxime/ceftazidime and fluoroquinolone resistance amongst isolates from wastewater from four Irish farms.

ESBL/ampC resistance										Fluoroquinolone resistance												
Mobile genetic element									AmpC promoter	Mobine genetic element				Mutational resistance identifiers								
Farm	Phenotypic AMR results	No. Isolates	*bla*_ACT- 14_	*bla*_CMY- 2_	*bla*_CTX-M- 14_	*bla*_CTX- M-15_	*bla*_CTX- M-65_	*bla*_SHV-12_	53bp_dC12T	*oqx*B	*qnr*B1	*qnr*B2	*qnr*S1	*gyr*A_ pS83L	*gyr*A_ pD87N	*par*C_ pA56T	*par*C_ pS80I	*par*C_ pE84G	*par*C_ pE84K	*par*E_ pL416F	*par*E_ pS458T	*par*E_ pS458A
1	CTX	8	0	0	3	3	0	0	2	1	1	0	0	0	0	0	0	0	0	0	0	0
	CTX / CIP	2	0	0	0	1	0	1	0	0	0	0	2	0	0	0	0	0	0	0	0	0
	CTX / CIP + NAL	1	0	1	0	0	0	0	0	0	0	0	0	1	1	0	1	0	0	0	1	0
	CIP + NAL	11	0	0	0	0	0	0	0	0	0	0	0	11	11	3	7	2	4	0	0	2
2	CTX	2	0	0	0	2	0	0	0	0	0	0	0	0	0	0	0	0	0	0	0	0
	CTX / CIP	2	0	0	0	2	0	0	0	0	0	0	2	0	0	0	0	0	0	0	0	0
	CTX / CIP + NAL	3	0	0	0	1	0	0	0	0	0	0	1	3	3	0	3	0	0	0	0	3
	CIP + NAL	4	0	0	0	0	0	0	0	0	0	0	1	4	4	2	4	0	0	0	0	2
3	CTX	3	0	0	1	0	0	0	2	0	0	0	0	0	0	0	0	0	0	0	0	0
	CTX / CIP	1	0	0	0	0	1	0	0	0	0	0	1	0	0	0	0	0	0	0	0	0
	CTX / CIP + NAL	6	0	0	0	0	0	0	5	0	0	0	0	6	6	1	6	0	0	0	0	4
	CIP + NAL	12	0	0	0	0	0	0	0	0	0	0	0	12	12	2	12	0	0	2	0	1
4	CTX	16	1	2	14	0	0	0	0	0	0	0	0	0	0	0	0	0	0	0	0	0
	CAZ	1	0	0	0	0	0	1	0	0	0	1	0	0	0	0	0	0	0	0	0	0
	CTX / CIP + NAL	4	0	3	1	0	0	0	0	0	0	0	0	4	4	0	4	0	0	0	3	0
	CIP + NAL	6	0	0	0	0	0	0	0	0	0	0	0	6	6	1	6	0	0	2	0	0
Four farms	CTX	29	1	2	17	5	0	0	4	1	1	0	0	0	0	0	0	0	0	0	0	0
	CAZ	1	0	0	0	0	0	1	0	0	0	1	0	0	0	0	0	0	0	0	0	0
	CTX / CIP	5	0	0	0	3	1	1	0	0	0	0	5	0	0	0	0	0	0	0	0	0
	CTX / CIP + NAL	14	0	4	1	1	0	0	5	0	0	0	1	14	14	1	14	0	0	0	4	7
	CIP + NAL	33	0	0	0	0	0	0	0	0	0	0	1	33	33	8	29	2	4	4	0	5
	**Total**	**82**	**1**	**6**	**18**	**9**	**1**	**2**	**9**	**1**	**1**	**1**	**7**	**47**	**47**	**9**	**43**	**2**	**4**	**4**	**4**	**12**

CTX, Cefotaxime; CAZ, Ceftazidime; CIP, Ciprofloxacin; NAL, Nalidixic acid.

Phenotypic resistance to ciprofloxacin (CIP) was confirmed by WGS in all relevant isolates (*n* = 52). The five isolates phenotypically described as CTX and CIP resistant all harbored *qnr*S1 in addition to genes encoding CTX-M-15 (*n* = 3), CTX-M-65 (*n* = 1) and SHV-12 (*n* = 1). The 47 *E. coli* isolates phenotypically identified as CIP and nalidixc acid (NAL) resistant harbored double mutations in *gyr*A and all had at least one mutation in *par*C and 20 of these also had at least one mutation in *par*E (Table 1). In addition, *qnr*S1 was identified in two *E. coli* isolates from farm 2 with CIP and NAL resistance phenotype (MIC value of 8 μg/mL for CIP and > 128 μg/mL for NAL) which also harbored point mutations in *gyr*A and *par*C and in *gyr*A, *par*C and *par*E, respectively.

In a few cases WGS identified resistance genes in isolates that did not display the corresponding phenotypic resistance. *K. pneumoniae* and *E. cloacae* were not phenotypically CIP resistant (MIC = 0.12 μg/mL for both) but carried *qnr*B1 and *qnr*B2 genes, respectively.

Less frequently, the resistance phenotype was not confirmed molecularly, as it was in the case of three CTX resistant isolates that did not contain corresponding resistance genes.

A total of 19 of all *E. coli* isolates showed co-resistance to cephalosporins plus fluoroquinolones. Eleven of these carried MGEs containing ESBL encoding genes, with 6 of the 11 also containing *qnr*S1 genes (Table 1). They originated from all four farms, mainly from farm wastewater, but four of them were from ICW treated water samples from farm 4.

### Identification of plasmid replicon types

**Figure 1:**
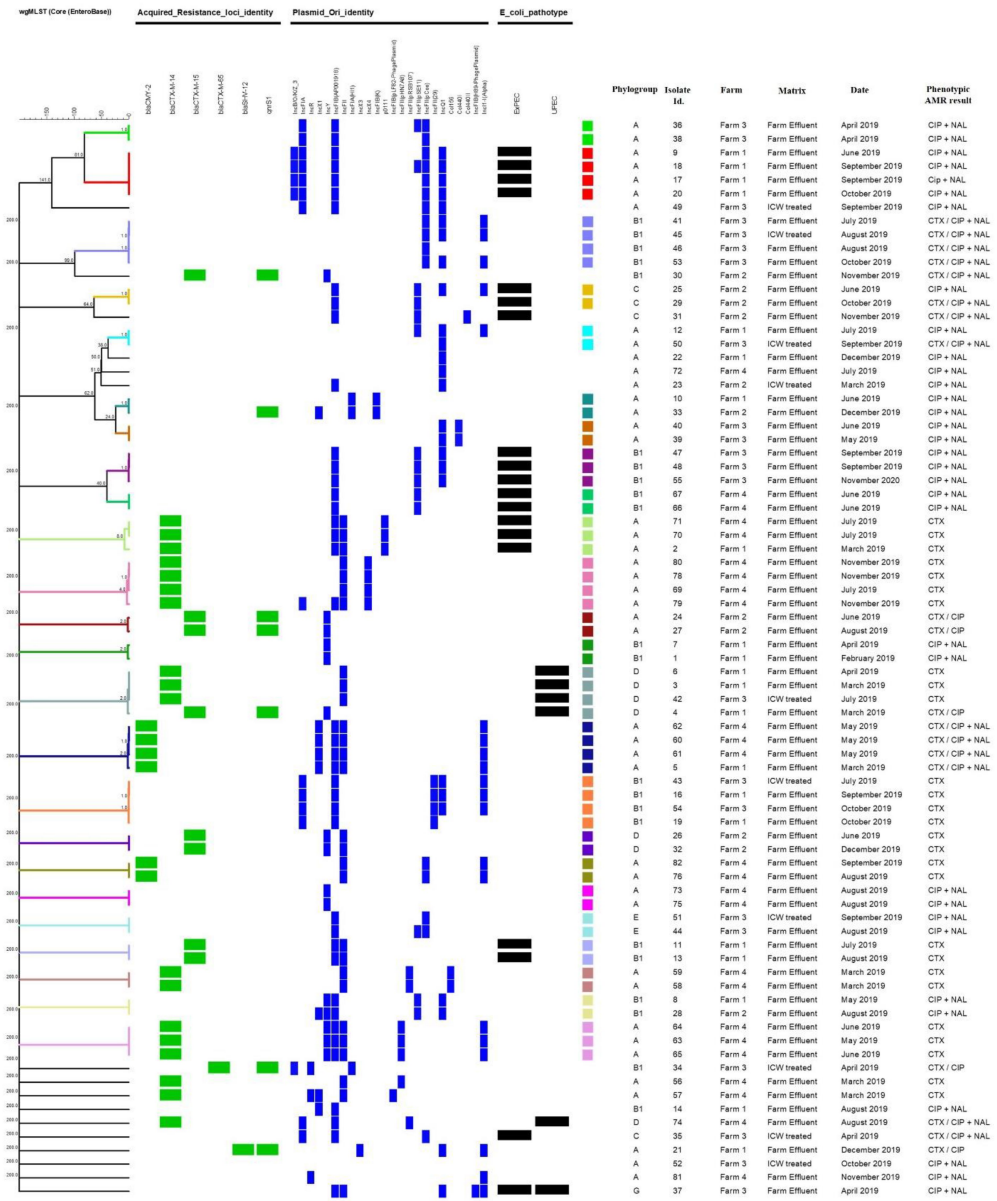
Dendrogram generated through cgMLST analysis with a scaling bar representing a phylogenic difference of 1 allele. The different color codes represent different clusters of highly related isolates (≤8 alleles). Also shown are the main acquired resistance genes, plasmids, pathoptyes and phylogroups identified. CTX, Cefotaxime; CIP, Ciprofloxacin; NAL, Nalidixic acid.

A total of 31 plasmid replicon types were identified amongst the 82 isolates with 24 identified in the 79 *E. coli* isolates (Figure 1). Multiple types were observed in the same isolate, with six plasmid replicon types identified in one *E. coli* isolate from farm 1 effluent and five identified in 11 different *E. coli* isolates originating from all four farm types including one from ICW-treated water. Among the 10 isolates from ICW-treated water samples, nine harbored between 1 and 5 plasmid replicon types. In contrast, there was one *E. coli* isolated from farm 3 ICW-treated water with a phenotypic AMR profile of CIP and NAL resistance, that did not harbor any plasmid replicon.

The most prevalent plasmid replicon type identified amongst the 82 isolates was IncFIB(AP001918), which was present in 41 (50%) of isolates. Four plasmid replicon types were only detected once which included two phage plasmids, i.e., IncFIB(pLF82-PhagePlasmid) and IncFIB(H89-PhagePlasmid) present in isolates from samples from two different farms (farms 3 and 4).

The most common replicon type amongst the isolates carrying *bla*_CTX-M-14_ was IncFII (present in 18/19 isolates) while the most common amongst isolates with genes encoding CTX-M-15 was IncY (present in 6/9 isolates). All six isolates harboring *bla*_CMY-2_ carried the IncFII plasmid replicon type; with four also carrying the IncX1 and IncFIB(AP001918) and the other two carrying IncFII(pCoo). The most abundant plasmid replicon types amongst the identified *qnr* positive isolates were IncY, IncR and IIncFIA(HI1), identified in 4, 3 and 3 isolates, respectively.

IncFII was more prevalent amongst the isolates identified as CTX/CAZ resistant but NAL sensitive, (80%) when compared with the isolates resistant to both CIP and NAL (3%). IncFIB(AP001918) prevalence was similar for both groups of isolates (43.3 and 60.5%).

Five plasmid replicon types were only detected in the *E. cloacae* isolate [IncHI2, IncHI2A, IncFIB(pECLA), IncFII(pECLA) and Col(pHAD28)], and two were confined to the *K. pneumoniae* isolate [IncFII(K) and IncFIA(HI1)].

### Identification of *E. coli* pathotypes within BioNumerics

Following analysis of the WGS data from the 79 *E. coli*, only two predicted pathotypes were identified amongst the eight possible pathotypes, i.e., 19 ExPEC and six UPEC as previously described (Malberg Tetzschner et al., 2020). While ExPEC was isolated from all four farms, the majority were isolated from farm 1 (7 isolates) followed by farms 3, 4 and 2 with five, four and two isolates, respectively. UPEC was isolated from farms 1, 3 and 4 only with three, two and one isolates, respectively. One of these isolates was defined as a hybrid of both ExPEC and UPEC and this was isolated from farm 3 (Figure 1).

### *In silico* and core genome (cg)MLST

*In silico* MLST typing yielded 22 different STs amongst the 79 *E. coli*, with ST10 the most common, identified in 20 isolates (Figure 1). Nineteen isolates of ST10 were from farm effluent samples from farm 1 (*n* = 6), farm 3 (*n* = 2) and farm 4 (*n* = 11), with only one of them from a ICW-treated sample (farm 3). ST744 was the next most common sequence type, identified in nine isolates from all four farms and two of these were isolated from ICW-treated water samples from farms 2 and 3 (Figure 1). Other sequence types identified in order of frequency were ST1431, ST162, ST69, ST1079, ST44, ST88, and ST48. In addition, two isolates classified as unknown STs were obtained from farm 2 farm effluent on two separate occasions during June and August 2019 (Figure 1). Each of the two unknown STs harbored *bla*_CTX-M-15_ and *qnr*S1 genes and both were identified as the same ST by the Pasteur and Whitman MLST schemes within BioNumerics, i.e., ST716 and ST872, respectively (data not shown). The 10 *E. coli* directly isolated from ICW-treated water samples were of 9 different STs with two of ST744 and others ST10, ST1011, ST1079, ST1431, ST2608, ST683, ST69, and ST88 (Figure 1).

When cgMLST was applied to the same 79 *E. coli* isolates, clusters both within and between farms were identified (Figure 1). Six of the 24 clusters had isolates from all four farms and all six had a difference of one or two plasmid replicon types (Figure 1). For example, isolates Id. 10 and 33 of ST744 were isolated from farms 1 and 2, respectively 6 months apart, both carried *qnr*S1 with one allele difference from each other by cgMLST but one carried an additional plasmid (IncX1).

In another cluster we observed isolates Id. 6 and 3 of ST69 from different sampling dates from farm 1 and isolate Id. 42 from farm 3, all the three were indistinguishable from each other and were shown to harbor *bla*_CTX-M-14_ and to carry the IncFII plamid. Another isolate (Id. 4) of the same ST was recovered from farm 1 that was 2 allele different and also differed by harboring the *bla*_CTX-M-15_ gene in addition to *qnr*S1 and by carrying the IncY plasmid (Figure 1).

### Determination of *E. coli* phylogroups (ClermonTyping)

**Figure 2:**
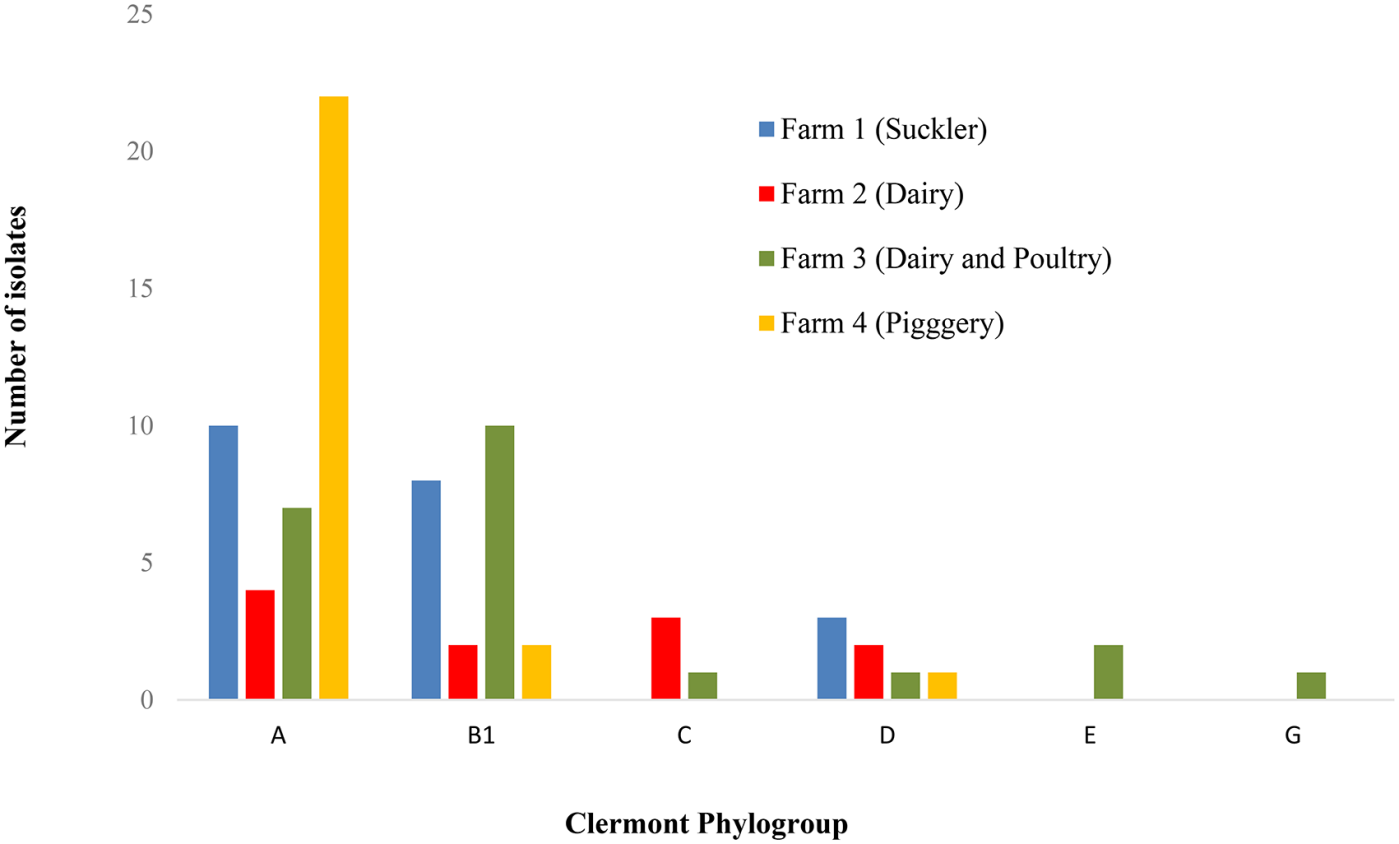
Distribution of different *E. coli* phylogroups amongst the four farms.

The 79 *E. coli* isolates were grouped into six phylogroups, i.e., A, B1, C, D, E and G with the majority (43 isolates) belonging to phylogroup A (Figures 1, 2) and these were mainly of ST10 (20 isolates) followed by ST744 (nine isolates). Phylogroup B1 was identified in 22 isolates and spread across eight different STs. Four phylogroups (A, B1, C and G) were identified among the 18 ExPEC (six different STs) and the five UPEC were all confined to phylogroup D and all were of ST69. The one isolate identified as ExPEC/UPEC was of phylogroup G (ST117). While phylogroup A and D were identified at all four farms, the majority of A originated from Farm 4 with no difference observed between farms for phylogroup D (Figure 2). Phylogroup C was confined to Farms 2 and 3 and phylogroups E and G were confined to Farm 3 (Figure 2).

### Identification of virulence genes and APEC pathotype

**Figure 3:**
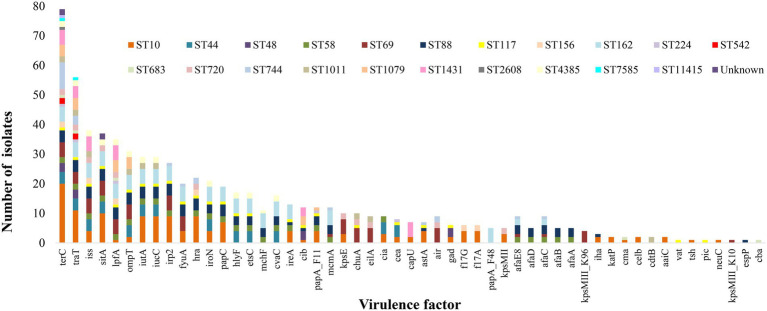
Distribution of virulence genes amongst the different ST of *E. coli* isolated from effluent and ICW-treated farm waste water. *terC*, Tellurium ion resistance protein; *tra*T, Outer membrane protein complement resistance; *iss*, Increased serum survival; *hra*, Heat-resistant agglutinin; *sit*A, Iron transport protein; *lpf*A, Long polar fimbriae; *omp*T, Outer membrane protease (protein protease 7); *iut*A, Ferric aerobactin receptor; *iuc*C, Aerobactin synthetase; *irp*2, High molecular weight protein 2 non-ribosomal peptide synthetase; *fyu*A, Siderophore receptor; *hra*, Heat-resistant agglutinin; *iro*N, Enterobactin siderophore receptor protein; *pap*C, Outer membrane usher P fimbriae; *hly*F, Hemolysin F; *ets*C, Putative type I secretion outer membrane protein; *mch*F, ABC transporter protein MchF; *cva*C, Microcin C; *ire*A, Siderophore receptor; *cib*, Colicin ib.; *pap*A_F11, Major pilin subunit F11; *mcm*A, Microcin M part of colicin H; *kps*E, Capsule polysaccharide export inner-membrane protein; *chu*A, Outer membrane hemin receptor; *eil*A, *Salmonella* HilA homolog; *cea*, Colicin E1; *cap*U, Hexosyltransferase homolog; *ast*A, EAST-1 heat-stable toxin; *air*, Enteroaggregative immunoglobulin repeat protein; *gad*, Glutamate decarboxylase; *f17*G, Adhesin subunit of F17 fimbriae; *f17*A, Subunit A of F17 fimbrial protein; *pap*A_F48, Major pilin subunit F48; kpsMII, Polysialic acid transport protein; Group 2 capsule; *afa*E8, Adhesin protein; *afa*D, Afimbrial adhesion; *afa*C, Outer membrane usher protein; *afa*B, Periplasmic chaperone; *afa*A, Transcriptional regulator; kpsMIII_K96, ABC-type polysaccharide/polyol phosphate export systems permease; Group 3 capsule; *ih*a, Adherence protein; *ka*tP, Plasmid-encoded catalase peroxidase; *cma*, Colicin M; *cel*b, Endonuclease colicin E2; *cdt*B, Cytolethal distending toxin B; *aai*C, Type VI secretion protein; *vat*, serine protease autotransporters of Enterobacteriaceae (SPATE); *tsh*, serine protease autotransporters of Enterobacteriaceae (SPATE); *pic*, serine protease autotransporters of Enterobacteriaceae (SPATE); *neu*C Polysialic acid capsule biosynthesis protein; *kps*MIII_K10, ABC-type polysaccharide/polyol phosphate export systems permease; Group 3 capsule; *esp*P, Putative exoprotein precursor; *cba*, Colicin B.

A total of 53 virulence genes were identified amongst the 79 *E. coli* isolates (Figure 3). The tellurium ion resistance protein encoding gene (*ter*C) was identified in all *E. coli*, followed by genes encoding for outer membrane protein complement resistance (*tra*T), increased serum survival (*iss*), iron transport protein (*sit*A), long polar fimbriae (*lpf*A), outer membrane protease (protein protease 7; *omp*T), ferric aerobactin receptor (*iuc*C), aerobactin synthetase (*iut*A), high molecular weight protein 2 non-ribosomal peptide synthetase (*irp*2) and siderophore receptor (*fyu*A), identified in 56, 38, 37, 34, 31, 29, 29, 27, and 27 isolates, respectively. The maximum number of virulence genes identified in one isolate was 25, and the minimum was one, with an average of 9 virulence genes per isolate. Common patterns of virulence gene distribution were observed in isolates from the same ST with 13 virulence genes confined to one particular ST, i.e., *tsh* [serine protease autotransporters of *Enterobacteriaceae* (SPATE), *neu*C (polysialic acid capsule biosynthesis protein), plasmid-encoded catalase peroxidase (*kat*P), Endonuclease colicin E2 (*celb*), and *aai*C (type VI secretion protein)] were identified in isolates of ST10 only. The genes *kps*MIII_K96 and *kps*MIII_K10 (ABC-type polysaccharide/polyol phosphate export systems permease; Group 3 capsule) were confined to isolates of ST69 only, *vat* and *pic* [serine protease autotransporters of *Enterobacteriaceae* (SPATE) to isolates of ST117 and the major pilin subunit F48 (*pap*A_F48), cytolethal distending toxin B (*cdt*B), colicin B (*cba*) and putative exoprotein precursor (*esp*P)] were identified in one particular ST only, i.e., ST162, ST1011, ST683 and ST88 accounting for 5, 2, 1 and one isolates, respectively (Figure 3). The APEC pathotype was identified when isolates had three or more of the virulence genes *iss*, *iro*N, *hly*F, *omp*T, and *uit*A (Johnson et al., 2008).

## Discussion

Dissemination of AMR genes in the Irish environment has been largely unknown to date. Our previous study reported the presence of AROs in farm effluents and how ICWs were able to reduce the load of AROs in farm effluent. In this paper WGS results confirmed the mechanisms of resistance to cephalosporins and fluoroquinolones identified phenotypically in most of those isolates. Regarding class A β-lactamase genes, 28 of 30 (93%) presumptive ESBL genomes possessed at least one CTX-M type β-lactamase encoding gene, with *bla*_CTX-M-14_ and *bla*_CTX-M-15_ most prevalent. These results are significant considering the most frequently encountered CTX-M variants in clinical isolates in Europe are CTX-M-14 and CTX-M-15 (Livermore and Hawkey, 2005; Canton et al., 2012; Irrgang et al., 2017; Sarno et al., 2021). The isolates encoding these *bla*_CTX-M_ genes were isolated from both farm effluent and ICW treated water, with *bla*_CTX-M-14_ mostly associated with the piggery (15/19) and *bla*_CTX-M-15_ with the dairy farm (5/9).

We identified six presumptive *E. coli* AmpC producers and all of them carried the *bla*_CMY-2_ gene, five of which were recovered from the pig farm and one from the beef farm. The increase in prevalence of *bla*_CMY-2_ conferring resistance to ceftiofur in pigs receiving a feed medicated with chlortetracycline and penicillin has been shown previously (Jahanbakhsh et al., 2015) and a recent study suggests that plasmid mediated rather than clonal spread likely play an important role for the emergence and transmission of *bla*_CMY-2_ between animals and humans (Ewers et al., 2021). While no *E. coli* isolates were identified as harboring the *bla*_CMY-2_ gene in the 22 isolates from farm 3, a recent study in Ireland reported that 13% of *E. coli* isolates from broiler farms harbored this gene (Byrne et al., 2022). While it was reassuring that no carbapenem resistance genes were found in any isolate in this study, it has been reported that AmpC enzymes promote carbapenem resistance in isolates with ESBL or defects in permeability (Mirsalehian et al., 2014; van Boxtel et al., 2016).

In the livestock and animal sectors, CTX-M-1, CTX-M-14 and CTX-M-15 ESBL types have frequently been detected in a number of different countries in Europe (Day et al., 2016; Ewers et al., 2021), as well as the United States (Afema et al., 2018), Canada (Cormier et al., 2019), and New Zealand (Collis et al., 2022). Agersø et al. (2011) also reported *bla*_CTX-M-14_ and *bla*_CTX-M-15_ in Danish pigs and pork with the highest prevalence was for *bla*_CTX-M-1_, in contrast with our results. The isolates from the study of Agersø et al. were further characterized (Hammerum et al., 2012) and they reported that pigs and pork can be a reservoir of ExPEC CTX-M-14-producing *E. coli*. We also identified ExPEC carrying *bla*_CTX-M-14_ in this study. Ewers et al. (2021) conducted a large-scale surveillance which included nine European countries, but not Ireland, where 2,993 commensal *Escherichia* spp. isolates were recovered from randomly collected fecal samples of healthy cattle, pigs and chickens in various abattoirs. By analyzing 99 isolates using WGS they identified *bla*_SHV–12_ (32.3%), *bla*_CTX–M–1_ (24.2%), and *bla*_CMY–2_ (22.2%) as the predominant ESBL/pAmpC types (Ewers et al., 2021). It is generally accepted that it is very difficult to compare countries and farms unless all studies are conducted the same, i.e., variations such as sample size, animal age, health status, variation in farming systems between countries, sample matrices and culture selection methods are all important variations to consider when comparing studies (Collis et al., 2022). This is why EFSA has recommended a joint European approach in order to address this knowledge gap (EFSA Biological Hazards (BIOHAZ) panel et al., 2021).

Although only encountered in one sample, the *bla*_CTX-M-65_ carrying *E. coli* isolate is of interest since it was from an ICW-treated water sample, and therefore it had the potential to enter waterways and the environment. CTX-M-65 has been previously referred to as a hybrid enzyme of both CTX-M-14 and CTX-M-15 with enhanced ESBL activity (He et al., 2015), and our isolate, in addition, also harbored *qnr*S1. A study in Bolivia showed that in the absence of selective pressure from antimicrobials, plasmid transfer of *bla*_CTX-M-65_ to other pathogens occurs frequently and is stable (Riccobono et al., 2015). In Europe, *bla*_CTX-M-65_ has also been reported in *S*. Infantis isolated from broilers and humans in Italy (Franco et al., 2015), in *E. coli* from cattle in the Netherlands (Dantas Palmeira and Ferreira, 2020) and more recently from beef and pork samples collected at retail in Portugal (Leão et al., 2021) and in *S*. Infantis from human cases in Spain (Vázquez et al., 2022). To the best of the authors’ knowledge, this is the first reported case of a *bla*_CTX-M-65_ isolated from the environment in Ireland.

While no carbapenemase or colistin resistant isolates were identified phenotypically, all isolates were screened for carbapenemase and colistin resistance genes and none were identified with the exception of the multidrug resistant (MDR) presumptive AmpC producing, and fluoroquinolone resistant *E. cloacae* isolated from the piggery that harbored an *mcr-9* gene (data not shown) in addition to *bla*_ACT-14_, *bla*_SHV-12_, *bla*_TEM-1B_ and *qnr*B2. The dissemination of MDR *E. cloacae* throughout the UK and Ireland has been previously reported and is now recognized as the third major nosocomial infection after *E. coli* and *K. pneumoniae* (Moradigaravand et al., 2016). The *bla*_ACT-14_ gene has been previously observed in *E. cloacae* isolates from dogs and wild birds (Literak et al., 2014; Boehmer et al., 2018). Our isolate also harbored the IncHI2-ST1 plasmid replicon; the family of IncHI2 plasmids often carry MDR genes that can be transferred horizontally along the food chain (Diaconu et al., 2021).

Plasmids encoding ESBLs and pAmpCs frequently harbor additional resistance genes and so can present a significant therapeutic challenge (Gupta and Bhadelia, 2014) and since some groups of antibiotics may persist in the environment for a very long time, i.e., fluoroquinolones have the lowest rate of degradation and highest resistance forming potential within the environment (Department of Health (DOH), 2017), it would be of interest to determine if the use of fluoroquinolones at farm level could increase the risk or persistence of resistant organisms and/or their resistance genes. Unfortunately, we did not have access to information on antimicrobial usage on any of the farms for this study. Lack of knowledge of antimicrobial usage at farm levels in Ireland has been previously highlighted (Martin et al., 2020) but will hopefully be the focus of future studies in our laboratory where we aim to examine the relationship between the use of on farm antibiotics and AMR. However, studies in the Netherlands and New Zealand (Hordijk et al., 2019; Collis et al., 2022) reported that antimicrobial usage only partially explained ESBL and AmpC positive samples and highlighted that factors other than antimicrobial usage may contribute to the transmission of AMR in the farm environment.

WGS also confirmed the phenotypic fluoroquinolone resistance observed and whether it was chromosomal, or plasmid mediated. While the presence of plasmid mediated genes has an additive effect and pose a higher risk of AMR dissemination, the low numbers of *qnr* genes in comparison to chromosomal resistance observed in this study is in agreement with other studies (Jacoby et al., 2003, 2006; Strahilevitz et al., 2009). Among isolates phenotypically resistant to CIP only and considered therefore presumptive plasmid mediated quinolone resistant isolates (Prendergast et al., 2022a), the most common gene found was *qnr*S1, present in seven isolates compared to *qnr*B1, *qnr*B2 and *oqx*B, which were each encountered in one instance only. Similar results were also observed in a study that reported *qnr*S followed by *qnr*B to be the most prevalent genes in *E. coli* from livestock and food in Germany (Juraschek et al., 2021). The *oqx*B gene was only observed in our study in the *K. pneumoniae* isolate phenotypically described as an ESBL producer susceptible to quinolones. While both *qnr* and *oqx* genes are plasmid mediated, *oqx* genes are members of the resistance-nodulation-division (RDN) efflux pump that have been shown to confer resistance to numerous antibiotics in addition to quinolones, including nitrofurantoin, quinoxalines, tigecycline, chloramphenicol, detergents, and disinfectants (Bharatham et al., 2021) and their presence has been shown to accelerate the development of fluoroquinolone resistance in *S*. Typhimurium (Wong et al., 2014).

All of the 47 presumptive chromosomal fluoroquinolone resistant isolates (resistant to both CIP and NAL) had multiple mutations in *gyr*A and/or *par*C, which have been associated with the expression of high-level fluoroquinolone resistance in clinical isolates (Ruiz et al., 1995; Minarini and Darini, 2012). In our previous study the MIC values were high, i.e., all 47 isolates displayed MIC values >128 μg/mL for NAL and ≥ 8 μg/mL for CIP. About one quarter of all isolates showed co-resistance to cephalosporins plus fluoroquinolones, which can be attributable to chromosomal mutation or acquisition of genes by horizontal transfer as explained by Tacão et al. (2014). Other researchers have shown that *qnr* genes can co-exist with the *bla*_TEM_ and *bla*_SHV_ alleles in clinical isolates and is most likely plasmid mediated (Jiang et al., 2008; Mood et al., 2015). Plasmid mediated resistance was observed in two isolates in our study with *E. cloacae* isolated from farm 4 effluent harboring *bla*_SHV-12_, *bla*_TEM-1B_ and *qnr*B2 (data not shown for TEM) and an *E. coli* isolate from farm 1 effluent that harbored *bla*_SHV-12_, *bla*_TEM-1B_ and *qnr*S1.

Amongst the 10 *E. coli* isolated from ICW-treated water, we identified *bla*_CTX-M-14_ in one isolate, *qnr*S and *bla*_CTX-M-65_ in another isolate and three isolates carried mutations in the AmpC promoter region in addition to mutations in *gyr*A and *par*C. In addition, double mutations in *gyr*A were identified in five of the isolates as well as single or double mutations in *par*C. The *E. coli* harboring *bla*_CTX-M-14_ was of predicted pathotype UPEC and one *E. coli* that carried a mutation in the AmpC promotor in addition to double mutations in *gyr*A and a single mutation in *par*C was of predicted pathotype ExPEC. The finding of AMR Enterobacterales along with MGEs from the ICW-treated water is concerning considering wildlife, arthropods, air and dust can act as vectors for short and long-distance environmental transmission of AMR contamination (EFSA Biological Hazards (BIOHAZ) panel et al., 2021). Resistant organisms and ARGs can enter groundwater through infiltration or bank filtration from surface water or leaching from the soil. This is of additional concern considering ground water is frequently used as a source of drinking water (Felis et al., 2020) and could also be absorbed by food crops, further contributing to exposure to and persistence of these contaminants (Department of Health (DOH), 2017).

The majority (54%) of our *E. coli* isolates grouped into phylogroup A, which was previously reported as the group where most human commensal strains worldwide belong to (Stoppe et al., 2017; Newman et al., 2021). The second most common phylogroup in our study was B1 (28%). No clear association between farms and phylogroups could be concluded, except for the isolates represented by phylogroup E which were confined to poultry, and this agrees with the study of Logue et al. (2017). Differences in phylogroup distribution have been observed previously and may be attributed to different circumstances such as geographical and climate location. A higher frequency of groups A and D from raw wastewater samples in Brazil was previously reported (Stoppe et al., 2017) whereas other studies of waste, ground and recreational waters reported higher frequencies of groups B2 followed by D in Australia (Anastasi et al., 2010), D followed by A in Poland (Mokracka et al., 2011) and A followed by B1 in Portugal (Figueira et al., 2011), the latter much the same as our results.

All five isolates identified as UPEC were grouped into phylogroup D and isolates within this group are considered of risk to public health (Orsi et al., 2007; Vázquez-Villanueva et al., 2023). While other studies reported that the predominant phylogroup in UPEC isolated from Europe and elsewhere is B2, followed by D (Dubois et al., 2010; Ejrnæs et al., 2011; Halaji et al., 2022), no isolate of phylogroup B2 was identified in this study. Since *E. coli* ST131 is considered an important emerging pathogen among phylogroup B2 strains harboring multiple AMR and virulence genes (Halaji et al., 2022), it was reassuring to note that there was no isolate of phylogroup B2 or ST131 identified in this study.

Isolates identified as ExPEC were not confined to one phylogroup, i.e., groups A (7 isolates), B1 (7 isolates) and C (four isolates). These findings therefore do not fully agree with Dale and Woodford (2015) and Sarowska et al. (2019), that indicated that the *E. coli* responsible for intestinal infections represent phylogenetic groups A, B1 or D and extraintestinal infections represent groups B2 and D.

Analysis of the MLST data revealed an association of certain STs with different farms, i.e., ST44 and ST48 were commonly associated with the piggery, ST58, ST156 and ST4385 with the beef farm, ST744 with the dairy and poultry farm and the two isolates recovered from the dairy farm were ST720. *E. coli* ST10 is recognized as an important human pathogen (Manges and Johnson, 2012) and have been previously isolated from poultry and pig sources (Bergeron et al., 2012). Among the 20 ST10 isolates in this study, 7 were of predicted ExPEC pathotype and this pathotype was also identified among *E. coli* isolates of ST88, ST162, ST58 and ST117. When the distribution of the major STs (ST10 and ST744) were compared between poultry and non-poultry farms, neither STs appeared to be overrepresented in a particular type of farms, although there was a larger proportion of ST744 among poultry vs. non-poultry farms than ST10. However, due to small numbers of isolates, i.e., 20 isolates of ST10 (3 poultry; 17 non-poultry) and nine isolates of ST744 (3 poultry and 6 non-poultry) it is difficult to make definitive conclusions, and this should be the focus of future studies.

ExPEC pathotypes were identified in all four farm types with the highest number identified in the beef farm (7 isolates) and the lowest in the dairy farm (3 isolates). This is concerning since ExPEC is a leading cause of urinary tract infections and responsible for the death of thousands of people every year especially young children (Munhoz et al., 2021). UPEC is the most common pathotype causing urinary tract infections (Guglietta, 2017; Rezatofighi et al., 2021; Hasan et al., 2022) and this pathotype was identified in six isolates and in three of the four farms with the highest number in the beef farm (3 isolates). ExPEC and UPEC can easily acquire MGEs and virulence factors from related bacteria and usually contain multiple pathogenicity islands (Johnson and Russo, 2005). Of interest to note was a ST117 isolate that was identified as a hybrid (ExPEC/UPEC) *E. coli* strain from the dairy and poultry farm which warrants further investigation regarding pathogenicity as they are considered to be more virulent (Santos et al., 2020; Munhoz et al., 2021). Of additional concern is that this isolate was grouped into phylogroup G. Isolates of this phylogroup have been identified as a poultry lineage with high virulence and antibiotic resistance potential and previous epidemiological data on 4,524 French and Australian strains suggested that phylogroup G of ST117 can also establish in humans and cause extra-intestinal diseases (Clermont et al., 2019). The APEC pathotype (presence of three or more of the genes *iss*, *iro*N, *hly*F, *omp*T, and *uit*A) was identified in 17 isolates with the majority identified at farm 4, piggery, (five isolates) with four isolates identified as APEC at each of the other three farms.

Plasmids play a critical role in the mobilization of AMR genes between bacteria and are one of the main reasons for resistance among Gram-negative bacteria (Li et al., 2019). In our study differences in plasmid replicon types in *E. coli* isolates from the same cluster that originated from different farms or from different sampling dates suggests local/regional transmission of plasmid replicons. We were however unable to confirm with the short-read sequencing that was used in this study if resistance genes were transferred by particular plasmids. More research is needed to decipher the role of particular plasmids in the transmission of AMR in the environment by undertaking full length sequencing of plasmids, and to better understand the ExPEC population and its role in AMR in the Irish farm environment.

More than 50 genes encoding virulence determinants, were identified, at different frequencies; *ter*C encodes a protein that functions in tellurium ion resistance and was identified in all 79 *E. coli* isolates, whereas other virulence genes were present in only one isolate. These virulence genes were associated with functions including stress, survival, regulation, iron uptake secretion systems, invasion, adherence and toxin production. The abundance of the tellurium ion resistance gene was surprising, as other studies have not reported such a high prevalence. A recent study of 167 *E. coli* genomes from human and animal environments circulating in 40 Brazilian cities identified all isolates as negative for *ter*C (Fuga et al., 2022). Other authors found that tellurium ion genes are mostly associated with STEC belonging to serotypes such as O26, O103, O111 O121, O145, and O45 (Lewis et al., 2018; Nguyen et al., 2021) none of which were isolated in our study (serotype data not shown). The gene *tra*T whose protein functions in outer membrane protein complement resistance, was the next most common virulence gene and was identified in 71% of isolates. It has been suggested that this gene may have a role in the pathogenesis of mastitis (Agüero et al., 1984), cystitis (Hasan et al., 2022) and pyelonephritis (Firoozeh et al., 2014). The serum survival gene *iss* and the *sit*A gene responsible for iron uptake, were identified in 46.7 and 43% of *E. coli* isolates, respectively, and they have been previously reported to be highly prevalent among avian pathogenic *E. coli* (Johnson et al., 2008; De Carli et al., 2015; Lima et al., 2019). In this study both of these genes were identified in isolates from all four farms without significant differences among them. In terms of pathogenicity, isolates identified as ExPEC, UPEC or hybrid (ExPEC and UPEC) had a larger prevalence of *sit*A and *iss* genes (alone or in combination) only in the beef farm (identified in 78.6% of isolates from the beef farm and 44.4, 40 and 41.6% at the dairy, dairy and poultry and pig farms respectively). No other association was observed in this study between STs, pathotypes, AMR types or species pathotypes and virulence genes.

In conclusion, to the best of the authors’ knowledge, this is the first study to provide insights into the prevalence of ESBL, AmpC and *qnr* genes in Enterobacterales collected from untreated and treated farm wastewater in Ireland. Our study shows that low levels of antimicrobial resistant Enterobacterales, persist even following wastewater treatment and that they are equipped with plasmids to transmit resistance genes of clinical relevance to the environment. This could ultimately lead to the contamination of drinking water or irrigation water used in crops. Further investigation on the role of plasmids in multiple antibiotic transfer mechanisms will be the focus of future studies in our laboratory.

## Data availability statement

Sequencing data generated for this study were deposited to the NCBI Sequence Read Archive (SRA) repository and can be found through the BioProject accession number PRJNA862318 https://www.ncbi.nlm.nih.gov/bioproject/PRJNA862318.

## Author contributions

DP, CB, DMo, and MG conceived the study. DMu and DJ performed WGS and uploaded sequences to NCBI. ÁO’D and JM contributed to provision of materials. DP analyzed data and wrote the manuscript with support from MG and RS. All authors contributed to the article and approved the submitted version.

## Funding

This work was carried out as part of the AREST project which is jointly funded by the Environmental Protection Agency, under the EPA Research Programme 2014–2020, and the Health Service Executive (2017-HW-LS-1). The EPA Research Programme is a Government of Ireland initiative funded by the Department of Communications, Climate Action and Environment. It is administered by the Environmental Protection Agency, which has the statutory function of co-ordinating and promoting environmental research.

## Conflict of interest

The authors declare that the research was conducted in the absence of any commercial or financial relationships that could be construed as a potential conflict of interest.

## Publisher’s note

All claims expressed in this article are solely those of the authors and do not necessarily represent those of their affiliated organizations, or those of the publisher, the editors and the reviewers. Any product that may be evaluated in this article, or claim that may be made by its manufacturer, is not guaranteed or endorsed by the publisher.

## Footnotes

^1^ http://clermontyping.iame-research.center/

^2^ https://cge.cbs.dtu.dk/services/MLST/
